# An Attitude Strength and Self-Perception Framework Regarding the Bi-directional Relationship of Job Satisfaction with Extra-Role and In-Role Behavior: The Doubly Moderating Role of Work Centrality

**DOI:** 10.3389/fpsyg.2016.00235

**Published:** 2016-03-03

**Authors:** Rene Ziegler, Christian Schlett

**Affiliations:** ^1^Fachbereich Psychologie, Universität TübingenTübingen, Germany; ^2^Institut für Psychologie, Universität FreiburgFreiburg, Germany

**Keywords:** job satisfaction, extra-role behavior, in-role behavior, work centrality, attitude strength, self-perception

## Abstract

Studies have identified variables either moderating the extent to which job satisfaction predicts work behavior or moderating the reverse impact of work behavior on job satisfaction. Based on an attitude strength and self-perception framework, we argue that certain variables may moderate both the predictive utility of job satisfaction for work behavior and the impact of work behavior on job satisfaction. Specifically focusing on work centrality, we hold that high work centrality renders job satisfaction a strong job attitude, whereas low work centrality renders job satisfaction a weak job attitude. Hence, the predictive utility of job satisfaction for both extra-role behavior and in-role behavior should be higher the more work is central to employees. In contrast, the influence of extra-role behavior, but not of in-role behavior, on job satisfaction should be higher the less work is central to employees. Results of a two-wave study (*N* = 176) were in line with these predictions. We discuss further variables that may play a similar role for the bi-directional relationship between job satisfaction and work behavior.

## Introduction

The relationship between employees’ job attitudes and their behavior on the job has been of long-standing interest in work and organizational psychology. Two prominent research streams concern the relationship of job satisfaction with in-role behavior (or task performance) and the relationship of job satisfaction with extra-role behavior (or citizenship behavior; Judge and Kammeyer-Mueller, 2012). In general, job satisfaction has been considered both as a determinant of work behavior and as determined by work behavior (Organ and Ryan, 1995; Judge et al., 2001; Riketta, 2008; Ng et al., 2009). Moreover, in regard to both directions of the job satisfaction-work behavior relationship, research has identified variables moderating the magnitude of the relationship. First, based on different perspectives some studies have investigated the extent to which various variables moderate the predictive utility of job satisfaction for work behavior. Second, further studies, based on yet other perspectives, have investigated whether certain variables moderate the influence of work behavior on job satisfaction. As a result, as has been noted (e.g., Judge et al., 2001), little theoretical integration of the accumulated literature has taken place.

Against this background, we propose a framework according to which certain variables may affect the magnitude of the job satisfaction-work behavior relationship in both directions. Specifically, drawing on social psychological attitude strength and self-perception research, we argue that several variables may serve to indicate whether employees’ job satisfaction represents a strong or weak attitude toward their job, and thus may influence the extent to which job satisfaction is predictive of work behavior as well as the extent to which work behavior impacts on job satisfaction. Based on this guiding framework, the present research tests the role of work centrality, the importance employees attach to work in general (Paullay et al., 1994), for the direction and size of the job satisfaction-work behavior relationship.

Specifically, research regarding the bi-directional relationship between attitudes and behavior indicates that strong attitudes are more predictive of future behavior than weak attitudes (Cooke and Sheeran, 2004; see also Schleicher et al., 2015). In this respect, we argue that job satisfaction represents a stronger attitude toward the job the more work is central for employees. Hence, we argue that job satisfaction is more predictive of both in-role behavior and extra-role behavior the more work is central for employees. Moreover, self-perception theory (Bem, 1972) holds that weak attitudes may be influenced by past behavior when the behavior was engaged in voluntarily (Bem, 1972; Holland et al., 2002). In this regard, a major difference between extra-role behavior and in-role behavior concerns the extent of employees’ discretion in showing the respective behavior. Whereas in-role behavior refers to behavior specified by role assignments and thus is prescribed and required from employees, engaging in extra-role behavior is at employees’ discretion (Katz, 1964). Therefore, we further argue that the impact of extra-role behavior, but not the impact of in-role behavior, on job satisfaction is higher the less work is central for employees.

Overall, the present research provides a novel framework regarding the bi-directional nature of the job satisfaction-work behavior relationship, suggesting that certain variables impact both the magnitude of the link from job satisfaction to work behavior and the magnitude of the link from work behavior to job satisfaction. Further, it is the first to consider work centrality as a moderator of the relationship between job satisfaction and work behavior. Finally, different from existing research showing that job satisfaction is a predictor of extra-role behavior, we aim to provide first direct evidence for the reverse impact of extra-role behavior on job satisfaction.

## Job Satisfaction and Work Behavior

Organizational functioning relies on employee behavior which is beneficial for an organization’s purpose. An important distinction with respect to facilitative work behavior concerns in-role behavior and extra-role behavior (Katz, 1964; Smith et al., 1983; Williams and Anderson, 1991; Van Dyne and LePine, 1998; Judge and Kammeyer-Mueller, 2012). In-role behavior refers to actions which are expected to be carried out by employees because of formal job descriptions and role assignments. Extra-role behavior, in comparison, is behavior which is not part of formal employment obligations but nonetheless facilitative of organizational effectiveness (Podsakoff et al., 2009). Most research on extra-role behavior has focused on organizational citizenship behavior (OCB; Organ, 1988). OCB refers to discretionary behavior of employees that is not explicitly recognized by formal reward systems and thus goes beyond focal role requirements (Organ, 1988). As meta-analyses have shown, in-role behavior and extra-role behavior are not only conceptually but also empirically distinct, albeit strongly correlated (Conway, 1999; Hoffman et al., 2007).

A plethora of research has studied the relationship of job satisfaction with work behavior. Indeed, meta-analyses have clearly established that job satisfaction is positively related to both in-role behavior and extra-role behavior (Organ and Ryan, 1995; Judge et al., 2001; LePine et al., 2002; Harrison et al., 2006; Ng et al., 2009). However, the relationships of job satisfaction with in-role behavior and extra-role behavior are of only medium size, and findings clearly suggest the existence of moderator variables affecting the size of these relationships (Judge et al., 2001; LePine et al., 2002). Moreover, with respect to the question of the direction of the relationship between job satisfaction and work behavior, it has been argued both that job satisfaction is a determinant of work behavior (e.g., Weiss and Cropanzano, 1996) and that work behavior is a determinant of job satisfaction (e.g., Porter and Lawler, 1968; Locke, 1970). In fact, research has identified variables moderating the size of the relationship in regard to both directions.

With respect to job satisfaction as a predictor of in-role behavior, for instance, Wright et al. (2007) have shown that the size of the relationship is moderated by personal well-being (Diener, 1984). In terms of the opposite direction, research has found that the extent to which in-role behavior predicts job satisfaction is affected, for instance, by self-esteem and job complexity (Korman, 1970; Baird, 1976; Inkson, 1978).

In regard to job satisfaction as a predictor of extra-role behavior, research has identified, for instance, conscientiousness (Bowling, 2010) and other orientation (Lester et al., 2008) to moderate the size of the relationship. With respect to extra-role behavior as a determinant of job satisfaction, in comparison, only suggestive evidence exists to date. For one, Tepper et al. (2004; see also Bowler and Brass, 2006) investigated the impact of *coworkers’ extra-role behavior* on *fellow employees’ job satisfaction* contingent on abusive supervision. Their research showed that coworkers’ extra-role behavior was related to positive job satisfaction change given low levels of abusive supervision, but was related to negative job satisfaction change given high levels of abusive supervision. Nonetheless, other research suggests that employees’ extra-role behavior might also have an impact on their own job satisfaction. First, it has been found that enacting extra-role behavior may improve employees’ affect (Glomb et al., 2011; but see also Somech and Drach-Zahavy, 2013; Bolino et al., 2014). Second, a number of studies have shown that affect at work impacts job satisfaction (Thoresen et al., 2003; Ilies and Judge, 2004; Schlett and Ziegler, 2014). Thus, existing findings suggest that job satisfaction might also be influenced by extra-role behavior.

Overall, research shows that job satisfaction may both predict work behavior and may be impacted by work behavior. Indeed, this aligns with social psychological research regarding the bi-directional relationship of attitudes and behavior.

## Job Satisfaction as Attitude Toward the Job

Job satisfaction represents employees’ attitude toward their job (Brief, 1998; Brief and Weiss, 2002; Weiss, 2002). In general, attitudes may have cognitive, affective, and behavioral antecedents, and may have cognitive, affective, and behavioral consequences (Zanna and Rempel, 1988; Eagly and Chaiken, 1993). Hence, with respect to behavior in particular, this tripartite model holds that attitudes may not only predict future behavior but may also be impacted by past behavior. Indeed, research has shown that attitudes are both predictive of future behavior (Kraus, 1995) and influenced by past behavior (Eagly and Chaiken, 1993). Whereas a rationale for the well-known assumption of an attitude-to-behavior link is provided by Fishbein and Ajzen (1975; see also Brief, 1998; Judge et al., 2001; Judge and Kammeyer-Mueller, 2012), a rationale for the less widespread assumption of a behavior-to-attitude link is provided by self-perception theory (Bem, 1967, 1972). Moreover, research has demonstrated the important role of attitude strength for both the direction and the size of the relationship between attitude and behavior (Petty and Krosnick, 1995).

### Attitude Strength

Research has shown that attitudes differ in the extent to which they are durable and impactful (Eagly and Chaiken, 1998). Whereas strong attitudes are very durable and impactful, weak attitudes are less durable and impactful. Of particular interest in the present context, strong attitudes are presumed to better predict future behavior than weak attitudes (Krosnick and Petty, 1995; Cooke and Sheeran, 2004; Bassili, 2008; see also Glasman and Albarracín, 2006). Theoretically, this can be due to stronger attitudes being more accessible at any moment in time, or because stronger attitudes are more capable of biasing perceptions of the attitude object and the context in which the behavior is performed (Fazio, 1986). Notably, research has investigated several indicators of the strength of an attitude, such as attitude ambivalence, attitude certainty, and attitude importance (Petty and Krosnick, 1995). Worth noting, it has been clearly shown that each of the various indicators of attitude strength is best investigated in its own right. Specifically, though different strength indicators have similar consequences, they differ with respect to their specific antecedents and the processes underlying their consequences (for a review, see Eaton et al., 2008).

Indeed, beginning with the work by Schleicher et al. (2004), job satisfaction research has already investigated the role of a number of properties indicating the strength of employees’ attitude toward their job for the extent to which job satisfaction predicts work behavior (Ziegler et al., 2012a,b; Schleicher et al., 2015). For example, focusing on attitude ambivalence, Ziegler et al. (2012b) showed that job ambivalence moderates the relationship between job satisfaction and extra-role behavior. Job ambivalence denotes the coexistence of positive and negative evaluations of one’s job. Thus, employees with high job ambivalence simultaneously like and dislike their job (see also Thompson et al., 1995; Jonas and Ziegler, 2007), rendering job satisfaction a weak job attitude given high ambivalence, but a strong attitude given low job ambivalence. Indeed, Ziegler et al. (2012b) showed that job satisfaction measured at a first point in time was more predictive of extra-role behavior collected at a second point in time the less employees were ambivalent regarding their attitude toward the job. Relatedly, Ziegler et al. (2012a) showed that job satisfaction was predictive of in-role behavior given low job ambivalence, but not predictive of in-role behavior given high job ambivalence.

Thus, in line with attitude strength research, job satisfaction is more predictive of work behavior the more employees’ job attitude is, for example, low in ambivalence (Ziegler et al., 2012a,b) or high in attitude certainty (Schleicher et al., 2015). However, attitude strength research not only indicates that strong attitudes are more predictive of future behavior than weak attitudes. Rather, based on self-perception theory (Bem, 1967, 1972), attitude strength research has also provided evidence for the reverse direction of influence, that is, weak attitudes may be more prone to be affected by past behavior than strong attitudes.

### Self-Perception Theory

Self-perception theory holds that “self-descriptive attitude statements can be based on the individual’s observations of his own overt behavior” (Bem, 1967, p. 185), thus suggesting that an individual is sometimes functionally “in the same position as an outside observer, an observer who must necessarily rely upon those same external cues [i.e., behavior] to infer the individual‘s inner states [i.e., attitudes]” (Bem, 1972, p. 2). Importantly, self-perception theory postulates two boundary conditions for individuals to rely on their own behavior as a basis to infer their own attitude. First, individuals are assumed to consider the attitudinal implications of their behavior only “to the extent that internal cues are weak, ambiguous, or uninterpretable” (Bem, 1972, p. 2). Thus, self-perception processes should be more likely to impact on an individual’s attitude to the extent that the attitude is weak as compared to strong (i.e., internal cues to the attitude as the inner state are weak). Second, individuals are assumed to consider “the external stimulus conditions under which [the behavior] occurs” (Bem, 1967, p. 185). Specifically, one’s own previous behavior is assumed to be indicative of one’s attitude only when engagement in the behavior was based on free choice instead of forced. Indeed, research has established that the impact of past behavior on attitudes depends on attitude strength and behavior voluntariness (e.g., Chaiken and Baldwin, 1981; for a review, see Olson and Stone, 2005).

Moreover, Holland et al. (2002) provided evidence that attitude importance moderates both the extent to which an attitude predicts future behavior and the extent to which it is impacted by past behavior. Attitude importance is defined as “an individual’s subjective sense of the concern, caring, and significance he or she attaches to an attitude” (Boninger et al., 1995, p. 160). According to Krosnick (1989, p. 297), “important attitudes are those that individuals attach personal importance to and care deeply about.” As has been shown, attitude importance is yet another indicator of attitude strength, with high importance rendering attitudes more predictive of behavior than low importance (e.g., Krosnick, 1988). Combining such findings with assumptions of self-perception theory, Holland et al. (2002) conducted a study in which participants’ attitudes toward Greenpeace as well as the importance of Greenpeace were measured at a first point in time. One week later, participants were provided with an opportunity to donate money to Greenpeace and were then asked again for their attitudes toward Greenpeace.

In line with predictions, participants’ attitudes toward Greenpeace at time 1 were more predictive of their decision to donate or not to donate money the more Greenpeace was important to them (i.e., the more positive their attitudes were the more likely they decided to donate). In other words, attitudes toward Greenpeace were a better predictor of future behavior for participants attaching high importance to Greenpeace as compared to participants attaching low importance to Greenpeace.

In contrast, controlling for time 1 attitudes, participants’ donation decision was more predictive of their time 2 attitudes toward Greenpeace the less Greenpeace was important to them (i.e., donating led their attitudes toward Greenpeace at time 2 to become more positive than their attitudes toward Greenpeace were at time 1). In other words, past behavior was more strongly related to changes in attitudes toward Greenpeace for participants attaching low importance to Greenpeace as compared to participants attaching high importance to Greenpeace.

## An Attitude Strength and Self-Perception Framework for the Magnitude and Direction of the Job Satisfaction-Work Behavior Relationship

We suggest that combining insights from attitude strength and self-perception research may provide a valuable general framework for understanding the bi-directional relationship of job satisfaction with work behavior. More specifically, we hold that the extent to which job satisfaction is predictive of future behavior as well as the extent to which job satisfaction is impacted by past behavior may depend on one and the same of the various indicators of the strength of employees’ job attitude. For instance, extending existing findings regarding the prediction of future behavior (Schleicher et al., 2004, 2015; Ziegler et al., 2012a,b), past behavior may impact job satisfaction more the lower attitude certainty or the higher job ambivalence.

However, we believe that an attitude strength and self-perception framework may also elucidate the role of constructs of longer-standing interest to the field of work and organizational psychology. In particular, we suggest that variables such as work centrality, job involvement (Paullay et al., 1994), and organizational identification (Ashforth and Mael, 1989) may have implications for the strength of job satisfaction as employees’ attitude toward the job. The focus of the present research is on work centrality, which has been of interest in work and organizational psychology since the early work by Dubin (1956) on work as a central life interest. Hence, in the following we specifically outline the role of work centrality for the job satisfaction-work behavior relationship, and return to job involvement and organizational identification in the discussion.

### Work Centrality and Attitude Strength

Work centrality has been defined “as the beliefs that individuals have regarding the degree of importance that work plays in their lives” (Paullay et al., 1994, p. 225; see also Kanungo, 1982). Employees for whom work is highly central care deeply about work. Employees considering work not as central, in comparison, care less about work. It has been found that work centrality is negatively related to the probability of quitting work after winning a lottery (Arvey et al., 2004) and turnover intentions (Bal and Kooij, 2011), and positively related to work engagement (Bal and Kooij, 2011) and affective organizational commitment (Moser and Schuler, 1993; Hirschfeld and Feild, 2000).

However, of greater importance in the present context are findings regarding the relationship of work centrality with job satisfaction, and of work centrality with work behavior. With respect to the relationship of work centrality with job satisfaction, as a matter of fact, findings are mixed, with some studies showing them to be positively related (e.g., Moser and Schuler, 1993; Mannheim et al., 1997; Cohrs et al., 2006; Bal and Kooij, 2011), and other studies showing them to be unrelated (e.g., Cohrs et al., 2006). Indeed, theoretically, work centrality and job satisfaction need not be positively related. Albeit employees may perceive work in general as an important component of their life, they may be rather dissatisfied with their present job. In contrast, employees may attach little value to work in general, but nonetheless be quite satisfied with their present job. In general, just as employees for whom work is central may be more or less satisfied with their present job, so may employees for whom work is not central be more or less satisfied with their present job. In regard to the relationship of work centrality with in-role behavior as well as extra-role behavior, similarly, findings are mixed with studies showing either a positive relationship (Mannheim et al., 1997; Ucanok, 2009) or no relationship (Diefendorff et al., 2002; Blakely et al., 2005). Taken together, then, existing research provides only inconclusive evidence for the assumption of work centrality as an antecedent of job satisfaction and work behavior.

In fact, we argue that it may be more appropriate to conceive of work centrality as a moderator of the job satisfaction-work behavior relationship rather than as an antecedent of job satisfaction and work behavior. In this regard, we hold that work centrality bears clear resemblance to attitude importance. More specifically, just as attitude importance indicates the importance of an attitude object (Krosnick, 1989; Boninger et al., 1995), so does work centrality indicate the importance of work (Paullay et al., 1994). Thus, from an attitude strength perspective, work centrality may be understood as an indicator of attitude importance. Hence, we argue that work centrality indicates whether job satisfaction is a strong or weak job attitude. In particular, high work centrality should render job satisfaction a strong and thus impactful attitude, whereas low work centrality should render job satisfaction a weak and thus less impactful attitude (Eagly and Chaiken, 1998).

### Research Aims and Hypotheses

Based on the assumption that work centrality indicates the strength of job satisfaction as employees’ job attitude, we propose that work centrality moderates both the utility of job satisfaction in predicting work behavior and the impact of past work behavior on job satisfaction. More specifically, for employees considering work as central, job satisfaction should be predictive of work behavior. Employees with a negative job attitude (i.e., dissatisfied with their job) should show less work behavior than employees with a positive job attitude (i.e., very satisfied with their job). For employees considering work not as central, in comparison, job satisfaction should be less predictive of work behavior. In other words, for whom work is not central, it should matter less whether job satisfaction is high or low. Overall, then, we posit that job satisfaction should be more predictive of work behavior given high rather than low work centrality. This should hold with respect to both extra-role behavior and in-role behavior. Further, we posit that this holds for job satisfaction as a predictor of concurrent work behavior (i.e., cross-sectional) as well as future work behavior (i.e., longitudinal).

*Hypothesis 1a*: Work centrality moderates the cross-sectional relationship of job satisfaction with extra-role behavior so that job satisfaction is more predictive of synchronous extra-role behavior the more work is central to employees.

*Hypothesis 1b*: Work centrality moderates the cross-sectional relationship of job satisfaction with in-role behavior so that job satisfaction is more predictive of synchronous in-role behavior the more work is central to employees.

*Hypothesis 2a*: Work centrality moderates the longitudinal relationship of job satisfaction with extra-role behavior so that job satisfaction is more predictive of future extra-role behavior the more work is central to employees.

*Hypothesis 2b*: Work centrality moderates the longitudinal relationship of job satisfaction with in-role behavior so that job satisfaction is more predictive of future in-role behavior the more work is central to employees.

**FIGURE 1:**
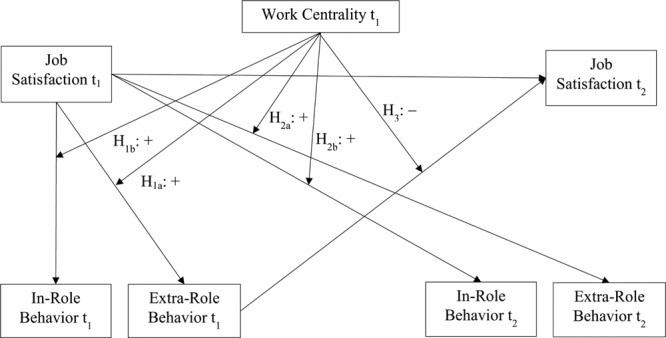
**Hypothesized effects of work centrality on the cross-sectional and longitudinal relationships of job satisfaction at Time 1 with extra-role behavior and in-role behavior, and on the longitudinal relationship of extra-role behavior at Time 1 with job satisfaction at Time 2**.

The above hypotheses refer to job satisfaction as a predictor of work behavior (also see **Figure 1**). However, work centrality may also influence the extent to which job satisfaction is impacted by past behavior. In particular, based on self-perception theory (Bem, 1967, 1972) and respective findings (Holland et al., 2002) we argue that job satisfaction may be impacted by past work behavior when the requirements outlined in self-perception theory for self-perception processes to occur are met. First, past work behavior should be more likely to influence job satisfaction when job satisfaction represents a weak job attitude (Bem, 1972). As argued, this should be the case when work is not central to employees. These employees may consult their own past behavior as a basis to infer their job satisfaction. Employees considering work as central, in comparison, may not rely on their own past behavior to infer the extent to which they are (dis)satisfied with their job because their job satisfaction represents a strong job attitude. However, second, for past work behavior to impact job satisfaction, work behavior should be of a voluntary nature (Bem, 1972), as is the case for extra-role behavior but not in-role behavior (Katz, 1964; Organ, 1988). Hence, we submit that past extra-role behavior should influence job satisfaction more the less work is central for employees. Past in-role behavior, in contrast, should be inconsequential for job satisfaction because self-perception theory holds that required behavior is not considered as indicative of one’s attitude. Based on this line of reasoning, we further posit (cf. **Figure 1**):

*Hypothesis 3*: Work centrality moderates the longitudinal relationship of extra-role behavior with job satisfaction so that job satisfaction is more influenced by past extra-role behavior the less work is central to employees^1^.

## The Present Study

To test our hypotheses, we conducted a two-wave study. At a first point in time, participants responded to scales measuring their job satisfaction, extra-role behavior, in-role behavior, and work centrality. At a second measurement point, participants were asked again for their job satisfaction, extra-role behavior, and in-role behavior. We deliberately collected self-ratings rather than other-ratings of participants’ work behavior because self-perception theory emphasizes people’s observations of their own overt behavior (Bem, 1967). However, self-ratings are often considered to be a deficient and inferior measurement method, and thus are held in low regard. More specifically, it has been argued that self-ratings may be biased by social desirability and self-enhancement motifs, and that common source variance may lead to artificially inflated relationships (Podsakoff et al., 2003). Yet, as Siemsen et al. (2010; see also Podsakoff et al., 2012; Carpenter et al., 2014) have shown, common source bias actually deflates regression-analytic interaction tests, as employed for testing the present hypotheses. Nonetheless, to control for socially desirable responding, we also collected participants’ responses to a social desirability scale.

### Methods

#### Participants and Procedure

Aiming at a heterogeneous sample, the study was conducted with employees of the German subsidiary of an international organization settled in the telecommunication sector. Each employee received an email from the human resource department containing a link to an online-questionnaire and information regarding voluntariness of participation, anonymity of responses, and approval by the industrial council. Participation at Time 1 was restricted to 2 weeks after initial email contact. The same procedure was employed for data collection at Time 2 which started 5 weeks after Time 1 data collection was finished. Participation at Time 2 was again restricted to 2 weeks. This time interval was chosen based on the assumption that data collection about 2 months apart is suitable for testing both hypothesized directions of the job satisfaction-work behavior relationship (Dormann and Zapf, 2001; Sturman et al., 2005; Riketta, 2008). In regard to the behavior-to-satisfaction link, in particular, we reasoned that employees may consider primarily more recent past behavior rather than temporally more distant behavior (e.g., a year ago). With respect to the predictive role of job satisfaction for future behavior, the present time lag was similar to prior longitudinal studies (e.g., Wanous, 1974; Bateman and Organ, 1983; Ziegler et al., 2012b). No monetary incentive or any other form of compensation for participating was offered to employees.

Out of 1447 employees contacted, 474 completed the questionnaire at Time 1. Of these, 176 also completed the questionnaire at Time 2.^2^ To test for non-random participant attrition, we referred to Goodman and Blum (1996). More specifically, we conducted a logistic regression analysis on “leavers” (i.e., Time-1 only participants) versus “stayers” (i.e., participants at Time 1 as well as at Time 2) with all variables measured at Time 1 as predictors (see below). No significant effect was found (all *p*s > 0.09). Thus, the probability of completing not only the Time-1 questionnaire but also the Time-2 questionnaire did not depend on the variables of interest in the present research. As Goodman and Blum (1996; p. 634) pointed out, finding no evidence of non-random sampling denotes that “researchers can be reasonably confident that attrition will not bias their subsequent longitudinal analyses of these variables.” With respect to the present research, hence, response bias is plausibly limited. Nonetheless, future work may examine this issue further.

One hundred and twenty-eight participants were male, 48 were female. Two percent of participants were younger than 26 years of age, 22% were between 26 and 35 years old, 53% were between 36 and 45, 21% were between 46 and 55, and 2% were older than 55 years of age. On average participants worked in their occupation for 14.26 (*SD* = 7.86) years. The majority of respondents (83%) held jobs without managerial responsibilities, 13% had responsibilities for work teams and 4% were responsible for other executive employees. Forty four of participants were working in technical departments, 35% in sales-related departments and 14% in administration (other departments: each below 2%). The majority of participants (93%) worked full-time, 4% worked more than two thirds part-time, 3% between one third and two thirds part-time, 1% less than one third part-time. Two percent of participants held time limited employment contracts.

### Measures

#### Job Satisfaction

Job satisfaction was measured with the Overall Job Satisfaction Scale (OJS; Brayfield and Rothe, 1951). The OJS consists of 18 items measuring satisfaction with one’s job in general (e.g., “My job is like a hobby to me”; “I feel fairly well satisfied with my present job”; “Each day of work seems like it will never end” – reverse scored). Responses to the items were taken on scales ranging from *does not apply at all* (coded as 1) to *applies completely* (5), and averaged so that higher scores indicate higher job satisfaction (Cronbach’s *α* = 0.92 and 0.93 for Time 1 and Time 2, respectively). We employed the OJS for two interrelated reasons. First, the OJS is balanced with respect to the affective and cognitive underpinnings of job satisfaction (Bowling and Hammond, 2008; Kaplan et al., 2009), thus representing a conceptually broad job satisfaction measure. Second, both in-role behavior and extra-role behavior represent broad classes of work behavior (Judge et al., 2001; LePine et al., 2002; Judge and Kammeyer-Mueller, 2012). Hence, to ensure correspondence in terms of construct generality (Fishbein and Ajzen, 1975), research on the relationship of job satisfaction with work behavior should employ broad measures of job satisfaction like the OJS (Fisher, 1980; Judge et al., 2001; Judge and Kammeyer-Mueller, 2012; Ziegler et al., 2012a).

#### Work Centrality

To measure work centrality, we employed the German work centrality scale validated by Moser and Schuler (1993; see also Lodahl and Kejner, 1965; Paullay et al., 1994), which consists of four items (e.g., “Most things in life are more important than work”; “I have other activities more important than my work”). Responses were made on scales ranging from *totally disagree* (coded as 1) to *totally agree* (7), and were averaged so that higher scores indicate higher work centrality (*α* = 0.71).

#### Work Behavior

To measure extra-role behavior and in-role behavior, participants’ responses to the FELA-S, a questionnaire validated by Staufenbiel and Hartz (2000), were collected. The questionnaire consists of 25 items toward which respondents are asked to indicate the extent to which each statement applies to them (1 = *does not apply at all* to 7 = *applies completely*). Based on the widespread questionnaire to measure OCB developed by Podsakoff et al. (1990), 20 items measure four of the five dimensions of OCB suggested by Organ (1988), that is, altruism (e.g., “I help others when they are overburdened with work”), conscientiousness (e.g., “I mind rules and regulations with utmost care”), sportsmanship (e.g., “I tend to make a mountain out of a molehill” – reverse scored), and civic virtue (e.g., “I keep myself informed about new developments in the company”). In line with findings by LePine et al. (2002), an overall score of extra-role behavior was calculated by averaging responses, with higher scores indicating more extra-role behavior (*α* = 0.78 and 0.81).

Furthermore, the FELA-S (Staufenbiel and Hartz, 2000) contains five items employed by Williams and Anderson (1991) to measure in-role behavior (“I adequately complete assigned duties”; “I fulfill responsibilities specified in the job description”; “I perform the tasks expected of me”; I meet the formal performance requirements of my job”; “I neglect aspects of my job I am obligated to perform” – reverse scored). Responses were averaged so that higher scores indicate more in-role behavior. At Time 1, internal consistency was *α* = 0.70; at Time 2 it was *α* = 0.62. The internal consistency at Time 2 was due to the last item of the scale (the only reverse coded item). Excluding it would have raised internal consistency to *α* = 0.77. We decided to retain the item for reasons of consistency with existing research employing the full five-item scale of in-role behavior (Staufenbiel and Hartz, 2000) which evinced satisfactory reliability at Time 1.^3^

#### Social Desirability

To control for socially desirable responding, participants were asked to respond to eight items of the social desirability scale developed by Stöber (2001). The scale consists of 17 items, of which 16 are scored (Stöber, 2001). However, for reasons of acceptance by the industrial council, we could only use those eight items with contents that were not deemed inappropriate in the context of the other questionnaire items referring to employees’ work-related perceptions (e.g., “I always eat a healthy diet”; “In traffic I am always polite and considerate of others”). For each remaining statement (e.g., “In conversations I always listen attentively and let others finish their sentences”; “When I have made a promise, I keep it - no ifs, and/or buts”) participants were prompted to indicate whether the statement applies to them (“true”) or not (“false”). Socially desirable responses were coded with 1, not socially desirable responses were coded with 0. Responses were averaged, with higher scores indicating higher social desirability. The internal consistency was relatively low, *α* = 0.60, reflecting the necessity to administer only half of the items and the forced choice format (Stöber, 2001, reported *alpha*s of the full 16-item scale of 0.74 and 0.75 in three studies).

### Data-Analyses

To test Hypotheses 1a,b and 2a,b, we conducted four hierarchical moderated regression analyses. Specifically, we analyzed extra-role behavior and in-role behavior at Time 1 (i.e., cross-sectional analyses) as well as at Time 2 (i.e., longitudinal analyses). In all analyses, in light of findings regarding their role for job satisfaction and work behavior (Snir and Harpaz, 2002; Ng and Feldman, 2008, 2010; O’Connell et al., 2011), we controlled for gender, age, tenure, and social desirability (all variables were centered; Aiken and West, 1991). Further, Time 1 job satisfaction and Time 1 work centrality were entered in a first step (both centered). At Step 2, the product of job satisfaction with work centrality was added to the regression model. In longitudinal analyses, finally, the respective work behavior at Time 1 was entered in a third step. The hypotheses led us to expect significant interactions at Step 2, showing that job satisfaction is more predictive of both concurrent and future work behavior given higher work centrality. It is important to note that we expected these interactions to be no longer significant when controlling for Time 1 work behavior at Step 3 of the longitudinal analyses because Hypotheses 2a,b do not refer to differences between the work behavior reported at Time 2 and the work behavior reported at Time 1. In other words, these hypotheses refer to the predictive utility of job satisfaction for the extent of future work behavior *per se* rather than changes in work behavior.

To test Hypothesis 3, we conducted a hierarchical moderated regression analysis on job satisfaction at Time 2, with age, gender, tenure, social desirability, extra-role behavior (Time 1), in-role behavior (Time 1), and work centrality entered in a first step (all centered). Further, at Step 2, the product of extra-role behavior with work centrality and the product of in-role behavior with work centrality were added to the regression model. Finally, job satisfaction at Time 1 was entered in a third step. The hypothesis led us to expect a significant interaction of extra-role behavior and work centrality in the final step, showing past extra-role behavior to have a higher impact on job satisfaction given lower work centrality. It is important to note that we did not expect this interaction to be significant at Step 2 because Hypothesis 3 refers to changes in job satisfaction, and thus necessitates controlling for job satisfaction at Time 1 (Step 3). In other words, this hypothesis refers to changes in job satisfaction relative to employees’ initial job satisfaction rather than the predictive utility of extra-role behavior for job satisfaction *per se*.

## Results

### Zero-Order Correlations Among Variables

As can be seen in **Table 1**, bivariate correlations were consistent with previous research. Specifically, job satisfaction was quite stable over the time period of the present study (Dormann and Zapf, 2001), as were extra-role behavior and in-role behavior (Sturman et al., 2005; Riketta, 2008). Also similar to previous findings (Hoffman et al., 2007), in-role behavior and extra-role behavior were positively correlated. Moreover, job satisfaction correlated positively with extra-role behavior as well as in-role behavior (Judge et al., 2001; LePine et al., 2002; Harrison et al., 2006; Ng et al., 2009), and work centrality was positively correlated with job satisfaction, extra-role behavior, and in-role behavior (Moser and Schuler, 1993; Mannheim et al., 1997; Cohrs et al., 2006; Ucanok, 2009; Bal and Kooij, 2011). Finally, social desirability was positively related to extra-role behavior ratings as well as in-role behavior ratings at Time 1.

**Table 1:** Range, means, standard deviations, and correlations.

	*Range*	*M*	*SD*	1	2	3	4	5	6	7	8	9	10
(1) Extra-role behavior (t_2_)	[1;7]	5.58	0.51										
(2) Extra-role behavior (t_1_)	[1;7]	5.60	0.50	0.84									
(3) In-role behavior (t_2_)	[1;7]	6.24	0.47	0.58	0.52								
(4) In-role behavior (t_1_)	[1;7]	6.28	0.52	0.53	0.59	0.70							
(5) Job satisfaction (t_2_)	[1;5]	3.53	0.59	0.43	0.42	0.35	0.27						
(6) Job satisfaction (t_1_)	[1;5]	3.60	0.56	0.42	0.45	0.34	0.31	0.88					
(7) Work centrality	[1;7]	3.48	1.16	0.31	0.32	0.25	0.20	0.52	0.48				
(8) Social desirability	[0;1]	0.83	0.19	0.26	0.29	0.07	0.20	0.11	0.08	0.12			
(9) Gender^a^	[-1;1]	–	–	-0.12	-0.11	-0.06	0.05	0.08	0.06	-0.09	-0.04		
(10) Age^b^	–	2.98	0.77	0.12	0.09	0.17	-0.01	-0.06	-0.02	0.00	0.01	-0.21	
(11) Occupational tenure	–	14.26	7.86	0.11	0.13	0.14	0.05	0.05	0.05	0.09	0.15	-0.15	0.73

*Correlations > | 0.14| are significant (*p* < .05). t_*1*_, measured at Time 1; t_*2*_, measured at Time 2. ^a^Gender was coded 1, female; -1, male; ^b^Age was measured in intervals (1 = less than 26 years; 2 = between 26 and 35 years; 3 = between 36 and 45 years; 4 = between 46 and 55 years; 5 = more than 55 years).*

### Factor Analyses and Common Method Bias

To test for the internal and discriminant validity of the measures, we conducted exploratory and confirmatory factor analyses (CFA). Parallel analyses (Horn, 1965) suggested retaining four factors of the exploratory factor analyses of the four measures at Time 1 (job satisfaction, extra-role behavior, in-role behavior, work centrality) as well as the four measures at Time 2 (job satisfaction, extra-role behavior, in-role behavior, social desirability). Furthermore, CFA were conducted that specified an unmeasured latent method factor with equal loading of all items at Time 1 (Podsakoff et al., 2003; Model 3a) and social desirability as a measured latent method factor with equal loading of all items at Time 2 (Podsakoff et al., 2003; Model 3b). Overall, CFA evinced good absolute model fits at both Time 1 (χ^2^/df = 1.60; RMSEA = 0.059) and Time 2 (χ^2^/df = 1.65; RMSEA = 0.061). Although the path coefficient of the unmeasured method factor at Time 1 was not significant (*p* = 0.133), a model without this factor yielded a somewhat worse fit (Δχ^2^ = 62.29, Δdf = 1, *p* < 0.001; RMSEA = 0.062). CFA at Time 2 revealed a significant relationship between the latent method factor social desirability and the items of the three other constructs (*B* = 1.56; *SE* = 0.46; *t* = 3.42; *p* < 0.001). These results indicate that participants responded in a social desirable manner, and thus suggest controlling for social desirability in the following analyses. Most importantly, CFA showed that factor loadings on job satisfaction, extra-role behavior, in-role behavior were significant except for one item at Time 1 (one of the 20 items measuring extra-role behavior). Given that the measure of extra-role behavior has been validated (Staufenbiel and Hartz, 2000) and employed repeatedly in existing research, we retained all items for reasons of comparability.

### Job Satisfaction as a Predictor of Work Behavior

#### Extra-Role Behavior at Time 1

**FIGURE 2:**
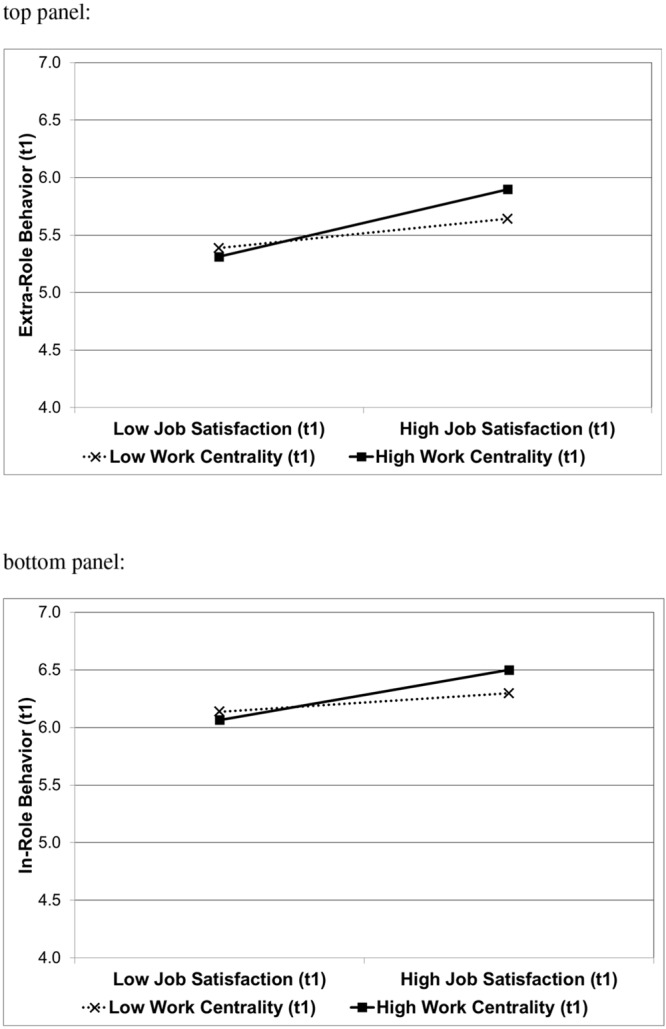
**Interactive effect of job satisfaction (Time 1) and work centrality (Time 1) on Time 1 extra-role behavior **(top panel)** and in-role behavior **(bottom panel)**.** Higher scores indicate more extra-role and in-role behavior. High and low job satisfaction, and high and low work centrality, refers to scores one standard deviation above and below the respective mean.

The first step of the regression analysis revealed that job satisfaction and social desirability were significantly related to extra-role behavior, whereas work centrality was unrelated to extra-role behavior (see **Table 2**, left half). More important, in line with Hypothesis 1a, the second step revealed the predicted job satisfaction by work centrality interaction (see **Figure 2**, top panel), explaining additional 3% of the variance of extra-role behavior (Cohen’s *f*^2^ = 0.053). Simple slope tests were conducted to determine the relationship of job satisfaction with extra-role behavior given high work centrality (i.e., one standard deviation above the sample mean) and low work centrality (i.e., one standard deviation below the sample mean; cf. Aiken and West, 1991). Job satisfaction was strongly related to extra-role behavior given high work centrality [*B* = 0.52; *SE* = 0.09; *t*(168) = 6.12; *p* < 0.001]. Given low work centrality, in comparison, job satisfaction was less strongly related to extra-role behavior [*B* = 0.23; *SE* = 0.08; *t*(168) = 2.94; *p* < 0.01].^4^

**Table 2:** Results of hierarchical moderated regression analyses on Time 1 extra-role behavior and in-role behavior (cross-sectional analyses).

	Extra-role behavior Time 1				In-role behavior Time 1			
	Step 1		Step 2		Step 1		Step 2	
(Step) Predictors	*B (SE)*	*t*	*B (SE)*	*t*	*B (SE)*	*t*	*B (SE)*	*t*
Constant	5.60 (0.03)	175.52^∗∗∗^	5.56 (0.03)	164.05^∗∗∗^	6.28 (0.04)	169.69^∗∗∗^	6.25 (0.04)	156.85^∗∗∗^
(1) Gender	-0.11 (0.07)	-1.53	-0.11 (0.07)	-1.50	0.06 (0.09)	0.68	0.06 (0.09)	0.73
(1) Age	0.05 (0.06)	0.85	0.05 (0.06)	0.83	-0.01 (0.07)	-0.06	-0.01 (0.07)	-0.10
(1) Tenure	-0.00 (0.01)	-0.16	-0.00 (0.01)	-0.21	0.00 (0.01)	0.19	0.00 (0.01)	0.15
(1) Social Desirability	0.67 (0.18)	3.77^∗∗∗^	0.63 (0.17)	3.63^∗∗∗^	0.46 (0.21)	2.27^∗^	0.43 (0.20)	2.14^∗^
(1) Job satisfaction (JS)	0.36 (0.07)	5.40^∗∗∗^	0.38 (0.07)	5.81^∗∗∗^	0.25 (0.08)	3.27^∗∗^	0.27 (0.08)	3.49^∗∗∗^
(1) Work centrality (WC)	0.04 (0.03)	1.16	0.04 (0.03)	1.25	0.03 (0.04)	0.70	0.03 (0.04)	0.75
(2) JS × WC			0.13 (0.04)	2.98^∗∗^			0.11 (0.05)	2.08^∗^
*R^2^*	0.30^∗∗∗^		0.33^∗∗∗^		0.13^∗∗∗^		0.15^∗∗∗^	

*^∗∗∗^*p* < 0.001; ^∗∗^*p* < 0.01; ^∗^*p* < 0.05.*

#### In-role Behavior at Time 1

In the first step, job satisfaction and social desirability turned out to also be significantly related to in-role behavior, whereas work centrality was unrelated to in-role behavior (see **Table 2**, right half). More important, in line with Hypothesis 1b, the second step revealed the predicted interaction of job satisfaction and work centrality, which explained an additional 2% of the variance of in-role behavior (Cohen’s *f*^2^ = 0.026). As illustrated in **Figure 2** (bottom panel), simple slope tests showed that job satisfaction was strongly related to in-role behavior given high work centrality [*B* = 0.39; *SE* = 0.10; *t*(168) = 3.85; *p* < 0.001], whereas job satisfaction was not significantly related to in-role behavior given low work centrality [*B* = 0.14; *SE* = 0.09; *t*(168) = 1.58; *p* = 0.12].

#### Extra-Role Behavior at Time 2

**FIGURE 3:**
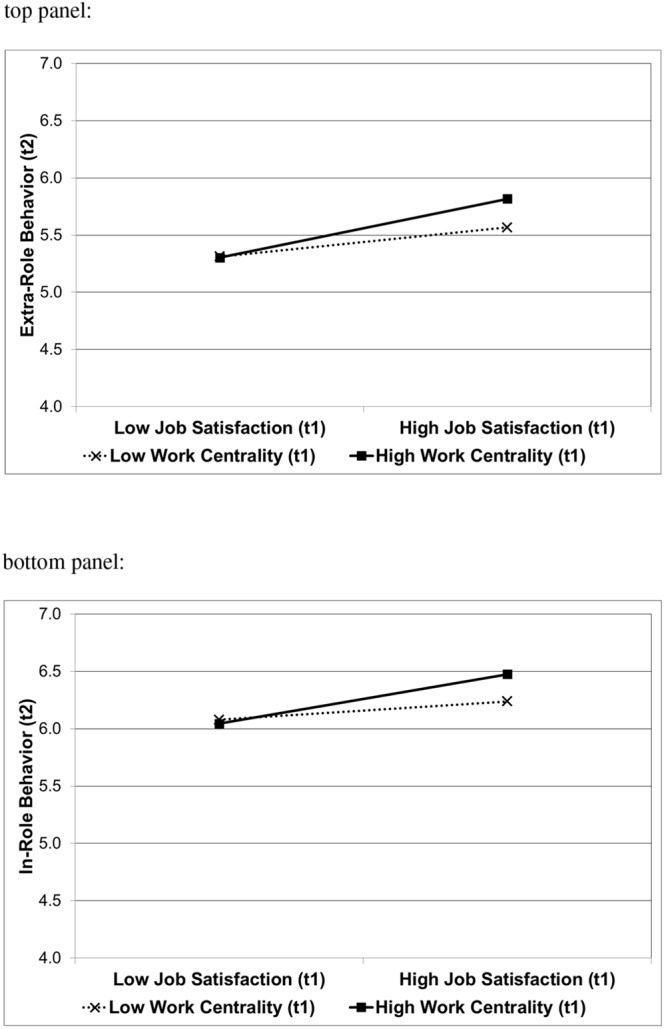
**Interactive effect of job satisfaction (Time 1) and work centrality (Time 1) on Time 2 extra-role behavior **(top panel)** and in-role behavior **(bottom panel)**.** Higher scores indicate more extra-role and in-role behavior. High and low job satisfaction, and high and low work centrality, refers to scores one standard deviation above and below the respective mean.

The first step of the longitudinal analysis showed that job satisfaction and social desirability were significantly related to future extra-role behavior. Work centrality, in contrast, was unrelated to extra-role behavior (see **Table 3**). As predicted (Hypothesis 2a), the job satisfaction by work centrality interaction was revealed in the second step, explaining additional 2% of the variance in extra-role behavior at Time 2 (Cohen’s *f*^2^ = 0.028). As illustrated by **Figure 3** (top panel), simple slope tests showed that job satisfaction was strongly related to extra-role behavior given high work centrality [*B* = 0.46; *SE* = 0.09; *t*(168) = 5.02; *p* < 0.001], whereas job satisfaction was less strongly related to extra-role behavior given low work centrality [*B* = 0.23; *SE* = 0.08; *t*(168) = 2.74; *p* < 0.01]. Finally, the third step showed that extra-role behavior at Time 1 was a strong predictor of extra-role behavior at Time 2, in fact the only significant effect remaining. Social desirability, job satisfaction, and the job satisfaction-work centrality interaction were no longer significantly related to extra-role behavior at Time 2.

**Table 3:** Results of hierarchical moderated regression analysis on extra-role behavior at Time 2 (longitudinal analysis).

	Extra-role behavior Time 2					
	Step 1		Step 2		Step 3	
(Step) Predictors	*B (SE)*	*t*	*B (SE)*	*t*	*B (SE)*	*t*
Constant	5.58 (0.03)	166.09^∗∗∗^	5.55 (0.04)	153.67^∗∗∗^	5.58 (0.02)	244.48^∗∗∗^
(1) Gender	-0.10 (0.08)	-1.34	-0.10 (0.08)	-1.30	-0.01 (0.05)	-0.21
(1) Age	0.12 (0.07)	1.87^+^	0.12 (0.07)	1.86^+^	0.08 (0.04)	1.92^+^
(1) Tenure	-0.01 (0.01)	-1.06	-0.01 (0.01)	-1.11	-0.01 (0.01)	-1.50
(1) Social desirability	0.63 (0.19)	3.38^∗∗∗^	0.60 (0.18)	3.25^∗∗∗^	0.08 (0.12)	0.64
(1) Job satisfaction (JS)	0.33 (0.07)	4.71^∗∗∗^	0.34 (0.07)	4.96^∗∗∗^	0.03 (0.05)	0.65
(1) Work centrality (WC)	0.05 (0.03)	1.51	0.05 (0.03)	1.57	0.02 (0.02)	0.94
(2) JS × WC			0.10 (0.05)	2.19^∗^	-0.01 (0.03)	-0.20
(3) Extra-role behavior (Time 1)					0.83 (0.05)	16.02^∗∗∗^
*R^2^*	0.27^∗∗∗^		0.29^∗∗∗^		0.72^∗∗∗^	

***p < 0.001; *p < 0.05; ^+^*p* < 0.07.

#### In-role Behavior at Time 2

The first step of the longitudinal analysis showed that job satisfaction was also significantly related to future in-role behavior, whereas work centrality was unrelated to in-role behavior (see **Table 4**). Consistent with Hypothesis 2b, the second step revealed the predicted interaction of job satisfaction with work centrality, explaining an additional 3% of the variance of in-role behavior at Time 2 (Cohen’s *f*^2^ = 0.033). Simple slope analyses showed that job satisfaction was strongly related to in-role behavior given high work centrality (*B* = 0.39; *SE* = 0.09; *t* = 4.30; *p* < 0.001), whereas job satisfaction was less strongly related to in-role behavior given low work centrality (*B* = 0.14; *SE* = 0.08; *t* = 1.77; *p* = 0.079). **Figure 3** (bottom panel) illustrates these findings. Finally, the third step showed that in-role behavior at Time 1 was a strong predictor of in-role behavior at Time 2. The job satisfaction-work centrality interaction was no longer significant. However, job satisfaction remained a significant predictor of in-role behavior at Time 2, though its predictive utility was substantially lower than in the first two steps.

**Table 4:** Results of hierarchical moderated regression analysis on in-role behavior at Time 2 (longitudinal analysis).

	In-role behavior Time 2					
	Step 1		Step 2		Step 3	
(Step) Predictors	*B (SE)*	*t*	*B (SE)*	*t*	*B (SE)*	*t*
Constant	6.24 (0.03)	188.54^∗∗∗^	6.21 (0.04)	174.81^∗∗∗^	6.23 (0.03)	238.05^∗∗∗^
(1) Gender	-0.03 (0.08)	-0.44	-0.03 (0.08)	-0.39	-0.07 (0.06)	-1.20
(1) Age	0.12 (0.06)	1.89^+^	0.12 (0.06)	1.87^+^	0.13 (0.05)	2.64^∗∗^
(1) Tenure	-0.00 (0.01)	-0.42	-0.00 (0.01)	-0.46	-0.00 (0.01)	-0.77
(1) Social Desirability	0.10 (0.18)	0.53	0.07 (0.18)	0.37	-0.20 (0.14)	-1.46
(1) Job satisfaction (JS)	0.25 (0.07)	3.64^∗∗∗^	0.26 (0.07)	3.90^∗∗∗^	0.10 (0.05)	2.01^∗^
(1) Work centrality (WC)	0.04 (0.03)	1.29	0.04 (0.03)	1.36	0.03 (0.02)	1.15
(2) JS × WC			0.10 (0.05)	2.32^∗^	0.04 (0.03)	1.21
(3) In-role behavior (Time 1)					0.61 (0.05)	11.99^∗∗∗^
*R^2^*	0.16^∗∗∗^		0.19^∗∗∗^		0.56^∗∗∗^	

*^∗∗∗^*p* < 0.001; ^∗^*p* < 0.05; ^+^*p* < 0.07.*

### Impact of Work Behavior on Job Satisfaction

**FIGURE 4:**
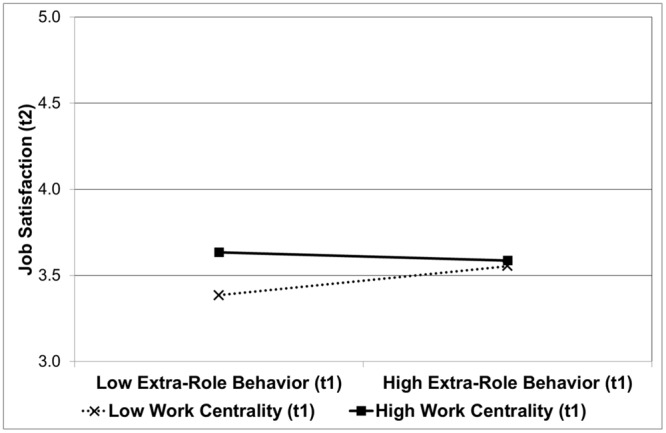
**Interactive effect of extra-role behavior (Time 1) and work centrality (Time 1) on job satisfaction at Time 2.** Higher scores indicate more job satisfaction. High and low extra-role behavior, and high and low work centrality, refers to scores one standard deviation above and below the respective mean.

The regression analysis on job satisfaction at Time 2 showed that work centrality as well as past extra-role behavior were significantly related to job satisfaction in the first two steps (see **Table 5**), whereas past in-role behavior was not. More important, in line with Hypothesis 3, the third step revealed that the interaction of extra-role behavior with work centrality was significant, explaining additional 0.6% of the variance of job satisfaction at Time 2 (Cohen’s *f*^2^ = 0.030). The interaction of in-role behavior with work centrality, in contrast, was not significant, as expected. Simple slope analyses were conducted to establish the relationship of extra-role behavior at Time 1 with job satisfaction at Time 2 given high work centrality (i.e., one standard deviation above the sample mean) and low work centrality (i.e., one standard deviation below the sample mean). As predicted, while past extra-role behavior was not significantly related to job satisfaction given high work centrality [*B* = -0.05; *SE* = 0.08; *t*(165) = -0.62, n.s.], past extra-role behavior was significantly related to job satisfaction given low work centrality [*B* = 0.17; *SE* = 0.08; *t*(165) = 2.09; *p* < 0.04]. **Figure 4** shows these findings.

**Table 5:** Results of hierarchical moderated regression analysis on job satisfaction at Time 2 (longitudinal analysis).

	Step 1		Step 2		Step 3	
(Step) Predictors	*B (SE)*	*t*	*B (SE)*	*t*	*B (SE)*	*t*
Constant	3.53 (0.04)	99.24^∗∗∗^	3.53 (0.04)	94.80^∗∗∗^	3.54 (0.02)	168.21^∗∗∗^
(1) Gender	0.18 (0.08)	2.19^∗^	0.19 (0.08)	2.22^∗^	0.07 (0.05)	1.37
(1) Age	-0.09 (0.07)	-1.35	-0.10 (0.07)	-1.35	-0.07 (0.04)	-1.63
(1) Tenure	0.01 (0.01)	1.03	0.01 (0.01)	1.02	0.01 (0.00)	1.37
(1) Social desirability	-0.13 (0.20)	-0.64	-0.13 (0.21)	-0.64	0.05 (0.12)	0.39
(1) Extra-role behavior (ERB)	0.38 (0.10)	3.97^∗∗∗^	0.38 (0.10)	3.92^∗∗∗^	0.06 (0.06)	1.06
(1) In-role behavior (IRB)	-0.01 (0.09)	-0.14	-0.00 (0.09)	-0.04	-0.05 (0.05)	-0.96
(1) Work centrality (WC)	0.22 (0.03)	6.72^∗∗∗^	0.22 (0.03)	6.47^∗∗∗^	0.06 (0.02)	2.95^∗∗^
(2) ERB × WC			0.05 (0.09)	0.57	-0.09 (0.05)	-2.03^∗^
(2) IRB × WC			-0.03 (0.08)	-0.36	0.03 (0.05)	0.63
(3) Job satisfaction (Time 1)					0.86 (0.05)	18.82^∗∗∗^
*R^2^*	0.38^∗∗∗^		0.38^∗∗∗^		0.80^∗∗∗^	

*^∗∗∗^*p* < 0.001; ^∗∗^*p* < 0.01; ^∗^*p* < 0.05.*

## Discussion

The present research tested hypotheses derived from an attitude strength and self-perception framework on the direction and size of the relationship between job satisfaction and work behavior. According to our framework, certain variables may influence when and to what extent job satisfaction allows predicting future behavior as well as when and to what extent past behavior may impact on job satisfaction. We argued that work centrality is one variable that may serve these roles. Specifically, exposing the conceptual similarities between work centrality (Paullay et al., 1994) and attitude importance as an indicator of the strength of an attitude (Boninger et al., 1995; Petty and Krosnick, 1995), we held that job satisfaction should represent a stronger job attitude the more employees consider work as central to their life (Dubin, 1956). Hence, in line with social psychological research on the attitude-to-behavior-link as a function of attitude importance (Krosnick, 1988; Holland et al., 2002), we predicted that job satisfaction should be stronger related to both in-role behavior and extra-role behavior the more employees consider work as central. Moreover, based on self-perception theory (Bem, 1967, 1972), we argued that extra-role behavior, but not in-role behavior, impacts more on job satisfaction the less employees consider work as central. Results of a two-wave study lent strong support to predictions.

Cross-sectional analyses showed that job satisfaction was stronger related to in-role behavior as well as extra-role behavior the more employees indicated to consider work as central. Similarly, longitudinal analyses revealed that job satisfaction was stronger related to both in-role behavior at Time 2 and extra-role behavior at Time 2 the more employees indicated to consider work as central.

Further, to our knowledge the current results are the first to clearly show that job satisfaction is not only a determinant of extra-role behavior (Organ, 1988; Weiss and Cropanzano, 1996; LePine et al., 2002), but rather that extra-role behavior may also influence job satisfaction (cf. Thoresen et al., 2003; Ilies and Judge, 2004; Glomb et al., 2011; Schlett and Ziegler, 2014). Analyses on job satisfaction at Time 2 showed that extra-role behavior at Time 1 had a more positive influence on job satisfaction the less employees considered work as central. In-role behavior at Time 1, in comparison, was unrelated to job satisfaction at Time 2 irrespective of the extent of work centrality.

In sum, higher work centrality rendered job satisfaction more predictive of concurrent as well as future work behavior. Lower work centrality, in comparison, rendered job satisfaction to be more impacted by past work behavior. Thus, as predicted from an attitude strength and self-perception framework, work centrality helped elucidate the bi-directional nature of the job satisfaction-work behavior relationship. Indeed, the present findings are the first to show that one and the same variable may serve to understand when job satisfaction predicts work behavior as well as when job satisfaction is impacted by work behavior.

Worth emphasizing in this regard, in line with our theorizing the current findings support an important difference with respect to the two directions of the job satisfaction-work behavior relationship. Employees setting high value on work in general were found to evince more future extra-role behavior as well as in-role behavior the more they were satisfied with their job. Thus, high work centrality renders job satisfaction to better predict future behavior, not to better predict behavior change. In regard to the behavior-to-job-satisfaction link, in contrast, we found that past extra-role behavior impacted on job satisfaction when employees set little value on work in general. Thus, low work centrality renders past work behavior to better predict job satisfaction change, not to better predict job satisfaction *per se*.

Worth noting, ascertaining that work centrality does not moderate the size of the relationship between in-role behavior and job satisfaction is not inconsistent with research showing that other variables moderate the extent to which in-role behavior influences job satisfaction (Porter and Lawler, 1968; Korman, 1970; Locke, 1970; Baird, 1976; Inkson, 1978). In fact, the present regression-analytic null-findings (i.e., in-role behavior was not predictive of job satisfaction, and work centrality did not moderate the relationship of in-role behavior with job satisfaction) are in line with our theoretical rationale according to which self-perception processes (Bem, 1967, 1972) should lead employees to consider their past work behavior as indicative of their job attitude only to the extent that the behavior was not prescribed. In this respect, in-role behavior is distinct from extra-role behavior. While in-role behavior is explicitly demanded by role assignments and job descriptions, extra-role behavior is of a more voluntary nature. However, for past work behavior to affect job satisfaction, work behavior needs not only to be unsolicited, but job satisfaction should also represent a weak job attitude (Holland et al., 2002). As we have argued and shown, this appears to be the case for employees who do not consider work as central.

Of further interest in this regard is, however, a comparison of results in the final steps of the longitudinal analyses. More specifically, job satisfaction (Time 1) was found to remain a significant predictor of in-role behavior at Time 2 when in-role behavior at Time 1 was controlled for (cf. **Table 4**). In comparison, in-role behavior (Time 1) was not found to predict job satisfaction at Time 2 when job satisfaction at Time 1 was controlled for (cf. **Table 5**). Thus, consistent with meta-analytic findings of panel studies regarding the causal relation between job satisfaction and in-role behavior (Riketta, 2008), the present cross-lagged analyses suggest that job satisfaction is more likely to influence in-role behavior than vice versa. With respect to the causal relation between job satisfaction and extra-role behavior, in contrast, a comparison of results of the present cross-lagged analyses (cf. **Tables 3** and **5**) does not suggest that job satisfaction is more likely to influence extra-role behavior rather than vice versa.

### Limitations

The internal consistency of the in-role behavior scale was relatively low at Time 2. This was due to the last item of the five-item scale developed by Staufenbiel and Hartz (2000), the only one with reverse coding. Although dropping it would have increased scale reliability substantially, we retained this item in our analyses for matters of comparability with the five-item scale at Time 1, showing satisfactory reliability, and existing research. Moreover, the two synchronous (i.e. cross-sectional) correlations of in-role behavior with job satisfaction, and of in role behavior with extra-role behavior, were highly similar, as were the respective two cross lagged correlations (cf. **Table 1**). Of further importance, rerunning all analyses without this item showed that all results were virtually identical to those reported herein. Hence, we hold that the present findings provide sound support for hypotheses.

Similarly, the restriction imposed by the industrial council to administer only that half of the items of the social desirability scale with content deemed appropriate in the current context of a questionnaire focusing on employees’ work-related perceptions led to less than satisfactory reliability of this measure (Stöber, 2001). However, concerns in this regard are mitigated by recent meta-analytic findings (Carpenter et al., 2014) showing that socially desirable responding may be less detrimental for the validity of self-ratings of work behavior and their relationships with variables of interest than previously assumed. Nonetheless, the present findings indicate the importance of scale length in further research on social desirability. Indeed, the Spearman–Brown prophecy formula predicts that the reliability of the full scale would have been α = 0.75 in the present study, and thus highly similar to reports of scale reliability by Stöber (2001).

We showed that work centrality moderates the extent to which job satisfaction predicts in-role behavior as well as extra-role behavior. Worth noting in this respect, although in-role behavior and extra-role behavior clearly covaried (cf. Hoffman et al., 2007), the correlations indicate some 60% of unshared variance of the two forms of work behavior. Hence, we hold that the empirical evidence on both types of facilitative work behavior represents non-redundant support regarding the moderating role of work centrality for the predictive utility of job satisfaction.

A related concern may refer to the similarities of the cross-sectional and longitudinal results involving the same type of work behavior. Specifically, in-role behavior as well as extra-role behavior were quite stable between the two points of measurement, the stability coefficients indicating that about 50% of the variance in in-role behavior at Time 2 is explained by the variance of in-role behavior at Time 1, and about 70% of the variance in extra-role behavior at Time 2 is explained by the variance of extra-role behavior at Time 1. Hence, certainly some overlap exists with respect to the cross-sectional and longitudinal analyses of each type of work behavior. Nonetheless, overall, we believe that the similar effects in the analyses on the predictive utility of job satisfaction for both concurrent and future work behavior provide sound empirical support for the moderating role of work centrality, even more so since common method variance of self-ratings potentially attenuates interaction effects (Siemsen et al., 2010).

Indeed, though providing significant support for the hypotheses, the interactions of work centrality with job satisfaction, and of work centrality with extra-role behavior, explained little additional criterion variance. Concerning this matter, McClelland and Judd (1993, p. 388) pointed out that the “detection of statistically reliable interactions (…) explaining an appreciable proportion of the variation of the dependent variable will be difficult” for field research (as compared to experimental research) for statistical reasons.

Finally, in line with self-perception theory emphasizing people’s observations of their own overt behavior (Bem, 1967), the present research employed self-ratings of participants’ work behavior. As has been argued, self-ratings may be biased by social desirability and self-enhancement motifs. Notably, however, a recent meta-analysis (Carpenter et al., 2014) provides clear evidence regarding the use and validity of self-ratings. With respect to OCB, in particular, Carpenter et al. (2014, 564) suggest that “self-rated OCB is not only a viable method of measuring OCB but also that it may represent a preferred manner of measuring employee OCB.” First, in terms of Cohen’s (1988) standards, differences in mean self-ratings and other-ratings are small. Second, self-ratings and other-ratings evince similar relationships with common correlates of OCB (e.g., job satisfaction), and the incremental contribution of self-ratings in predicting various criteria when controlling for other-ratings is higher than vice versa. Further, the correlation of other-ratings of OCB with other-rated in-role behavior as compared to the correlation of self-ratings of OCB with self-rated in-role behavior indicates that other-ratings of work behavior may be affected more by halo error. Hence, “self-ratings of OCB should not be summarily deemed methodologically deficient because of concerns over substantial inflation bias, common method bias, or socially desirable responding” (Carpenter et al., 2014, p. 565). In view of these results and conclusions, we hold that self-ratings of work behavior are no less appropriate to measure work behavior than other methods, and well-suited for testing assumptions regarding the relationship of job satisfaction with work behavior. Nonetheless, it may be an interesting avenue for further research to also test the present hypotheses with other-ratings of work behavior.

### Practical Implications

Both in-role and extra-role behavior are of central importance for organizational effectiveness. In this regard, the present research suggests that taking actions to assure high job satisfaction may be conducive primarily for employees caring deeply about work in general. For employees caring less about work in general, in comparison, high job satisfaction is less conducive in promoting extra-role and in-role behavior. Hence, organizations may profit from securing high work centrality across their workforce, for instance by safeguarding against hiring applicants placing little emphasis on work in general.

### Implications for Future Research

An attitude strength and self-perception framework may also advance our understanding of the interplay of job satisfaction with other job attitude-related concepts in the literature. Job involvement, for example, has been found to interact with job satisfaction in predicting absenteeism (Wegge et al., 2007). Different from work centrality, which has also been dubbed work involvement (Kanungo, 1982), job involvement refers to “the degree to which one is cognitively preoccupied with, engaged in, and concerned with one’s *present job*” (Paullay et al., 1994, p. 225, italics added). Job involvement may also serve to indicate the strength of job satisfaction as job attitude. Specifically, job satisfaction may represent a stronger job attitude given higher job involvement. With respect to predicting work behavior, hence, job satisfaction may evince higher utility the more employees are involved in their job. Inversely, extra-role behavior may influence job satisfaction more the less employees are involved in their job.

Similarly, organizational identification (Ashforth and Mael, 1989; Riketta, 2005) has been investigated as a predictor of extra-role behavior (e.g., Van Dick et al., 2006). We deem it plausible to understand organizational identification as indicating the strength of job satisfaction as job attitude as well. More specifically, organizational identification refers to the extent to which membership in an organization is a central aspect of an employee’s self-concept (Ashforth and Mael, 1989), thus resembling the concept of ego involvement, which has been shown to also serve as an indicator of attitude strength (Petty and Krosnick, 1995; Thomsen et al., 1995). Hence, high organizational identification may also indicate that job satisfaction represents a strong job attitude, whereas low organizational identification indicates that job satisfaction represents a weak job attitude. Therefore, job satisfaction may be more predictive of in-role as well as extra-role behavior the more employees identify with their organization. Reversely, extra-role behavior may impact job satisfaction more the lower employees’ organizational identification. Overall, we believe an attitude strength and self-perception framework may afford intriguing insights in future research on the size and nature of the job satisfaction-work behavior relationship.

## Conclusion

Abundant research has clearly established a positive relationship between job satisfaction and work behavior (Judge et al., 2001; LePine et al., 2002; Harrison et al., 2006; Ng et al., 2009). Based on attitude strength research (Petty and Krosnick, 1995), we argued that work centrality indicates the extent to which job satisfaction represents a strong or weak job attitude. Indeed, job satisfaction was more predictive of two distinct types of facilitative work behavior, in-role behavior and extra-role behavior, the more employees considered work as central. Based on self-perception theory (Bem, 1972), we further argued that past extra-role behavior, but not past in-role behavior, may influence job satisfaction given low work centrality. In fact, the present results provide first evidence that extra-role behavior may influence job satisfaction. More generally, we outlined an attitude strength and self-perception framework aimed at advancing our understanding of the role of different variables for the bi-directional relationship between job satisfaction and work behavior. We hope such a framework enlivens future work on the perennial search for the “Holy Grail” of organizational psychology (Landy, 1989).

## Author Contributions

All authors listed, have made substantial, direct and intellectual contribution to the work, and approved it for publication.

## Conflict of Interest Statement

The authors declare that the research was conducted in the absence of any commercial or financial relationships that could be construed as a potential conflict of interest.

## Footnotes

1 To be clear, it is not the case that we argue that higher work centrality “is” a stronger job satisfaction. Rather, we hold that job satisfaction is a stronger attitude toward the job when work centrality is high. Hence, job satisfaction should affect work behavior more strongly when work centrality is high rather than low. Thus, two employees differing in work centrality (high vs. low) may have the same job satisfaction, but job satisfaction should affect work behavior more for the employee with high work centrality. Conversely, extra-role behavior should impact on job satisfaction more strongly when work centrality is low rather than high. Thus, two employees differing in work centrality (high vs. low) may have shown the same extra-role behavior in the past, but past extra-role behavior should impact on job satisfaction more for the employee with low work centrality.

2 The final participation rate (12% or 0.12) may appear low at first sight. However, response rates at any single point in time often figure between 0.3 and 0.4. Hence, the present rate, involving two measurement points in time, lies within this to-be-expected range (0.3 × 0.3 < 0.12 < 0.4 × 0.4).

3 All analyses reported in “Results” section revealed highly similar results when conducted without this item.

4 As suggested by Reviewer 1, we also tested our hypotheses by running weighted least-squares (WLS) models instead of ordinary least-square (OLS) models. To do so, we took the inverse of social desirability (to avoid division by zero: 1/(1+SD) as a regression weight in all analyses. Results were highly similar. In particular, the crucial interactions of work centrality with job satisfaction (in predicting extra-role and in-role behavior at Times 1 and 2) and the interaction of work centrality with extra-role behavior (in predicting job satisfaction at Time 2) all remained significant. We report OLS results not the least because “WLS involves one important cost relative to OLS regression (...). In WLS, measures such as *R*^2^ do not have a straightforward meaning” (Cohen et al., 2003, p. 147).
