# Supplementary material for: An Attitude Strength and Self-Perception Framework Regarding the Bi-directional Relationship of Job Satisfaction with Extra-Role and In-Role Behavior: The Doubly Moderating Role of Work Centrality
Source: Front Psychol. 2016 Mar 3;7:235. doi: 10.3389/fpsyg.2016.00235 (PMC4776305; doi:10.3389/fpsyg.2016.00235)
Supplement: Supplementary file 1 [file Table_1.DOCX]

Supplementary Material

An Attitude Strength and Self-Perception Framework Regarding the Bi-directional Relationship of Job Satisfaction with Extra-Role and In-Role Behavior: The Doubly Moderating Role of Work Centrality

Rene Ziegler*, Christian Schlett

*** Correspondence: Rene Ziegler**: rene.ziegler@uni-tuebingen.de

# Supplementary Table 1. *Logistic regression analyses and t-Tests (stayers versus leavers).*

|  | Logistic Regression | | | *t*-Tests | | |
| --- | --- | --- | --- | --- | --- | --- |
| Variables | *B* | *SE* | Wald | Mean difference | *SE* | *t* |
|  |  |  |  |  |  |  |
| Extra-Role Behavior | .15 | .24 | 0.36 | < 0.001 | 0.05 | < 0.001 |
| In-Role Behavior | -.12 | .20 | 0.33 | 0.01 | 0.05 | 0.20 |
| Job satisfaction | .19 | .20 | 0.83 | -0.02 | 0.06 | -0.40 |
| Work Centrality | -.18 | .11 | 2.66 | 0.13 | 0.10 | 1.36 |
| Gender | .16 | .23 | 0.47 | -0.04 | 0.04 | -0.94 |
| Age | -.22 | .13 | 2.80^+^ | 0.13 | 0.07 | 1.80^++^ |
| Tenure | -.001 | .002 | .12 | 5.67 | 8.67 | 0.65 |
| Constant | -.18 | 1.30 | .02 |  |  |  |
| *-2 log-Likelihood* | 613.09* |  |  |  | |  |
| *Model Chi-square* | 7.58 |  |  |  | |  |

*Notes*. Stayers = 0; Leavers = 1; Mean difference = stayers minus leavers; * *p* < .05; ^+^ *p* = .09; ^++^ *p* = .07.
